# Spatial distribution, work patterns, and perception towards malaria interventions among temporary mobile/migrant workers in artemisinin resistance containment zone

**DOI:** 10.1186/1471-2458-14-463

**Published:** 2014-05-17

**Authors:** Khin Thet Wai, Myat Phone Kyaw, Tin Oo, PeThet Zaw, Myat Htut Nyunt, Moe Thida, Thar Tun Kyaw

**Affiliations:** 1 Department of Medical Research (Lower Myanmar), No. 5 Ziwaka Road, 11191 Yangon, Myanmar; 2 Department of Health, National Malaria Control Programme, Nay Pyi Taw, Myanmar

**Keywords:** Malaria, Temporary mobile/migrant workers, Spatial distribution, Perceptions, EDPT, Bed net use, Epidemiological surveillance

## Abstract

**Background:**

Mobile populations are at a high risk of malaria infection and suspected to carry and spread resistant parasites. The Myanmar National Malaria Control Programme focuses on preventive interventions and vector control measures for the temporary mobile/migrant workers in Myanmar Artemisinin Resistance Containment Zones.

**Methods:**

A prospective cross-sectional study was conducted in 2012 in Kawthaung and Bokepyin townships of Tanintharyi Region, Myanmar, covering 192 mobile/migrant aggregates. The objectives were to identify the spatial distribution of the mobile/migrant populations, and to assess knowledge, attitudes, perceptions, and practices concerning malaria prevention and control, and their preferred methods of interventions. The structure of the192 migrant aggregates was investigated using a migrant mapping tool. Individual and household information was collected by structured interviews of 408 respondents from 39 aggregates, supplemented by 12 in-depth interviews of health care providers, authorities, volunteers, and employers. Data were analyzed by triangulating quantitative and qualitative data.

**Results:**

The primary reasons for the limitation in access to formal health services for suspected malaria within 24 hours were identified to be scattered distribution of migrant aggregates, variable working hours and the lack of transportation. Only 19.6% of respondents reported working at night from dusk to dawn. Among study populations, 73% reported a perceived risk of contracting malaria and 60% reported to know how to confirm a suspected case of malaria. Moreover, only 15% was able to cite correct antimalarial drugs, and less than 10% believed that non-compliance with antimalarial treatment may be related to the risk of drug resistance. About 50% of study population reported to seeking health care from the public sector, and to sleep under ITNs/LLINs the night before the survey. There was a gap in willingness to buy ITNs/LLINs and affordability (88.5% vs. 60.2%) which may affect their sustained and consistent use. Only 32.4% across all aggregates realized the importance of community participation in effective malaria prevention and control.

**Conclusions:**

Community-based innovative approaches through strong collaboration and coordination of multi-stakeholders are desirable for relaying information on ITNs/LLINs, rapid diagnostic test, and artemisinin combination therapy and drug resistance successfully across the social and economic diversity of mobile/migrant aggregates in Myanmar.

## Background

Despite declining morbidity and mortality related to malaria globally in the last ten years, malaria remains one of the major public health problems in Myanmar and a significant majorityof malaria cases were caused by *Plasmodium falciparum*[1,2]. The Greater Mekong Sub-region (GMS) is known as the epicenter of multi-drug resistant *P. falciparum,* and the presence of artemisinin resistant *P. falciparum* has been documented in Myanmar along with Cambodia and Vietnam. A gradual decline in the therapeutic efficacy of common artemisinin-based combination therapy [3] and the evidence of artemisinin resistance in the regions of Myanmar bordering Thailand [4], led to the endorsement of Myanmar Artemisinin Resistance Containment (MARC) strategy by World Health Organization. The MARC strategy, implemented by eight implementing partners of the National Malaria Control Program (NMCP) [5], focuses on the mobile migrant populations, with a major emphasis on improving access to vector control measures including personal protection, malaria diagnosis, antimalarial drugs and treatment.

The mobile populations are at an increased risk of exposure to malaria, and it is highly suspected that they are more likely than other groups to carry and spread resistant parasites [6]. In Myanmar a mobile migrant aggregate may comprise workers as well as their families including children, and seasonal migrants may frequently move from one place to the other, with a prolonged interval at times, based on the availability of work and/or security of their livelihoods [7,8]. The nature of their life style hampers with the acquisition of adequate health information and access to quality health care, placing them at a high risk of substandard drug, late diagnosis, inadequate treatment and insufficient follow up, all of which are considered to be contributing factors to the development of drug resistant malaria [9,10]. In addition, the acceptability of and compliance to antimalarial drug treatment may be influenced by different socio-economic factors and/or cultural and belief systems of the specific mobile group, as documented in Lao PDR and Cambodia [11]. There are no data, in our knowledge, describing the nature and distribution of mobile migrant populations along the southern border of Myanmar with Thailand (in Tanintharyi Region), and the structure and conditions of malaria interventions and health care facilities in the region.

## Methods

### Study design

A prospective cross-sectional descriptive study was conducted as part of the ongoing MARC survey. The primary objectives were to identify the spatial distribution of mobile/migrant aggregates in Tanintharyi Region, to evaluate their knowledge, attitudes, perceptions, and practices including, but not limited to, the use of insecticide-treated or long-lasting nets (ITNs/LLINs) and early diagnosis and prompt treatment (EDPT) of malaria. The study was also designed to explore the social and cultural preferences in access to malaria diagnosis and treatment, so as to recommend effective strategies for malaria interventions in the mobile populations in support of the MARC survey. The location, movement and distribution of the mobile populations were collected in connection with available health care facilities, using geospatial technology [12].

### Study site

**Figure 1:**
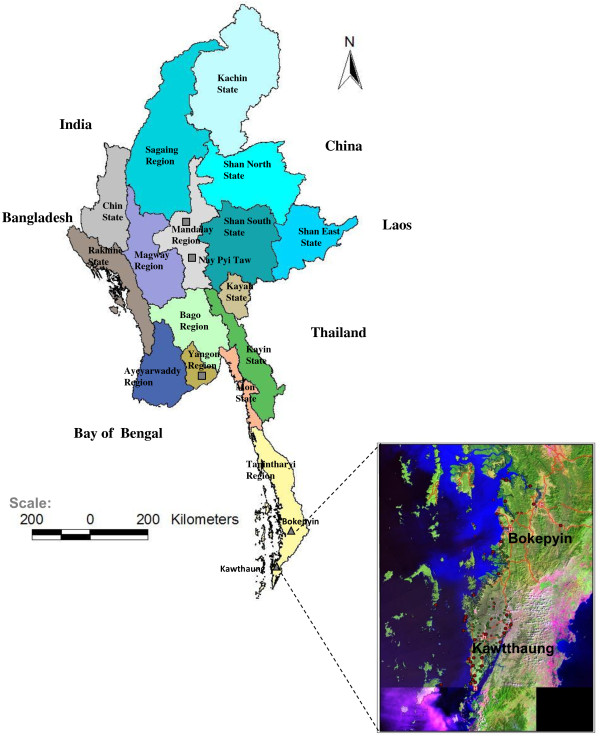
Map of the Republic of Union of Myanmar showing locations of Kawthaung and Bokepyin townships.

The study was conducted in Kawthaung and Bokepyin townships of Kawthaung district,Tanintharyi Region. The study sites are located in the southern-most costal region of Myanmar bordering Thailand (Figure 1), where the local climate alternates between a cool-dry (December-March) and hot and humid-wet season (April-November), with a heavy torrential rain falls in May-September, providing a perfect favorable ground for breeding of malaria vectors. The study site was selected based on a strong suspicion of artesunate resistance [4], and on undocumented knowledge of a high population movement.

### Study population and aggregates

Two types of populations were included in the study: migrant populations working temporarily in rubber plantations, palm oil plantations, fishing sites or a various types of fieldwork (internal migrants were those who traveled from one geographic region to other within Myanmar, and cross-border migrants were those who migrated across the Myanmar-Thailand border); and stable populations residing in the study site including basic health staff (BHS), local authorities, employers, volunteers and health providers. Mobile/migrant population was defined as a group of individuals (worker plus his/her family members) who had the following characteristics: 1) history of travel across the Myanmar-Thailand border or between the study site and other parts of the country; and 2) history of residence in the study site over a month or across the malaria transmission season, or for the whole year (stayed at least for one year). The migrant aggregates were categorized as “Large” for a group of at least 60 individuals whose economic activity was homogenous in nature and located far away from residing villages; “Small” for a group consisting of 25–60 individuals who lived close to economic activity but may or may not to be close to the village; and “Cut-off village settlement” who lived close to villages, share same resources and economically dependent on villages.

### Data collection methods and statistical consideration

All accessible aggregates of migrant workers in Kawthaung and Bokepyin townships, totaling 192 aggregates, were invited and agreed to participate in the study and included in the initial migrant mapping. From each aggregate, 20% of respondents were randomly selected for a structured interview. A targeted sample size was 408 respondents in total, with an assumption that 10% of the populations seek EDPT or used ITNs/LLINs within a specified time period, and a marginal error of 5% and 95% confidence level. One adult respondent from each family of participating migrants was consecutively interviewed, until the required sample size was reached. The mapping took about 40 days to cover 192 aggregates, the structured household interview 2–3 aggregates per day and 4–10 households were interviewed per aggregate in randomly selected 39 aggregates.

A mapping team was trained in the study aggregate mapping by the Myanmar Information Management Unit (MIMU) in the geospatial technology. The location of each mobile/migrant aggregate was treated as a spatial unit being marked by GARMIN e-Trex Geographical Positioning System (GPS) devices [13] and illustrated in Geographic Information System (GIS) based satellite maps from MIMU, applying ‘Migrant Mapping Tool’ (a recording form including geo-coordinates of each aggregate; see Additional files 1 and 2) introduced by International Organization of Migration (IOM) in Myanmar. Three to four key informants per aggregate joined the study interview. The interview questionnaires focused on economic activities, estimated population structure, and access to malaria care providers and on the assessment of specific strategies. A total of 408 structured-interviews, including 12 in-depth interviews (IDI), were conducted by using the structured-interview questionnaire that covered household and individual information (see Additional files 1 and 2). The guideline for the IDI was developed by Department of Medical Research (Lower Myanmar).

The accuracy and consistency of data were evaluated by thorough form checks and ensued by double data entry, and described in frequency distributions and cross tabulations of variables of interest. The SPSS version 17.0 software was used for analyzing quantitative data, and qualitative data were triangulated for meaningful interpretations.

#### Ethical consideration

The study was reviewed and approved by the Ethics Review Committee of the Department of Medical Research (Lower Myanmar). Informed consent was obtained prior to data collection, and the study was conducted in accordance with the declaration of Helsinki and in respect of the participant’s privacy and confidentiality.

## Results

**Figure 2:**
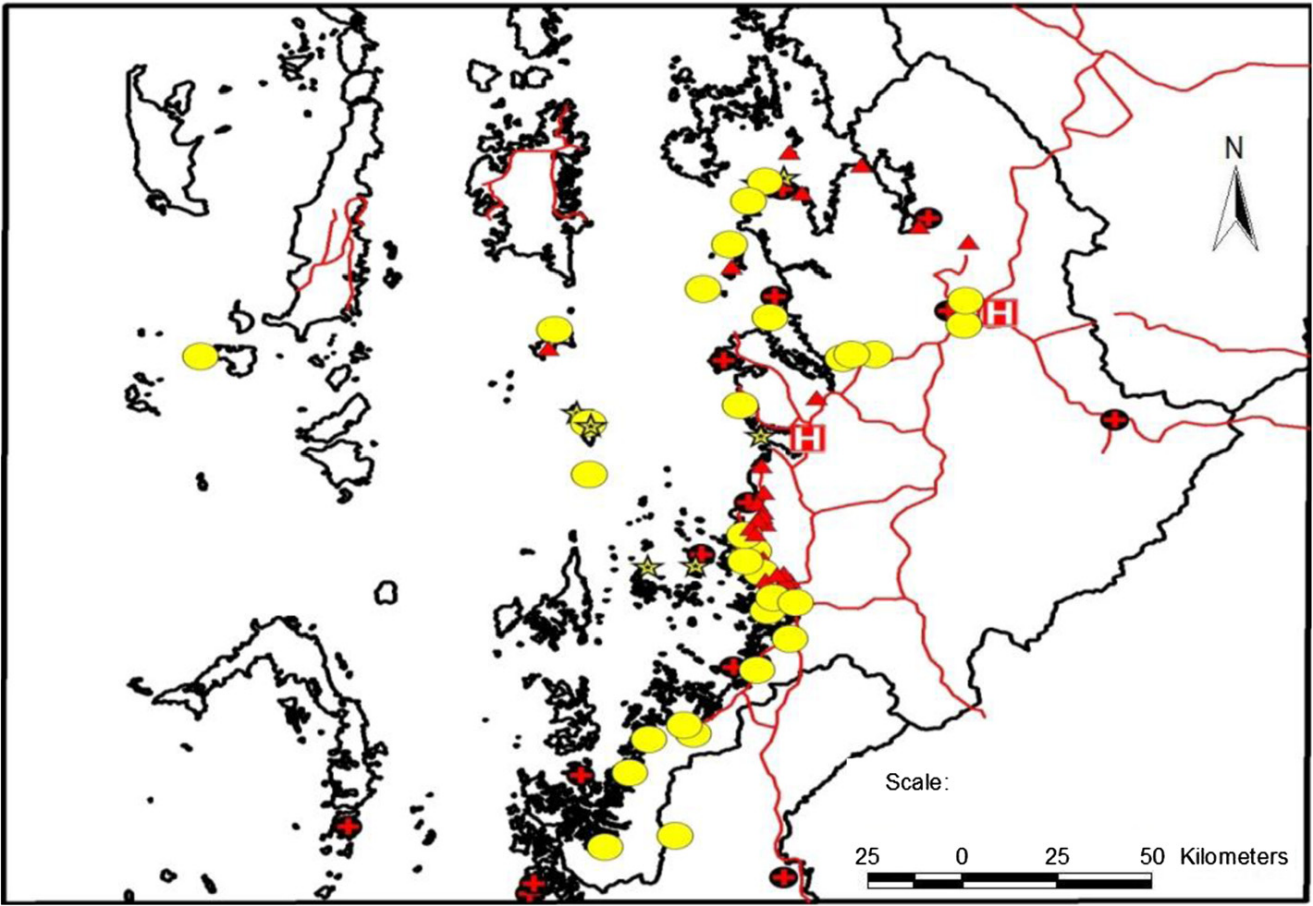
Spatial distribution of aggregates of temporary migrant workers and public health facilities (Bokepyin township).

**Figure 3:**
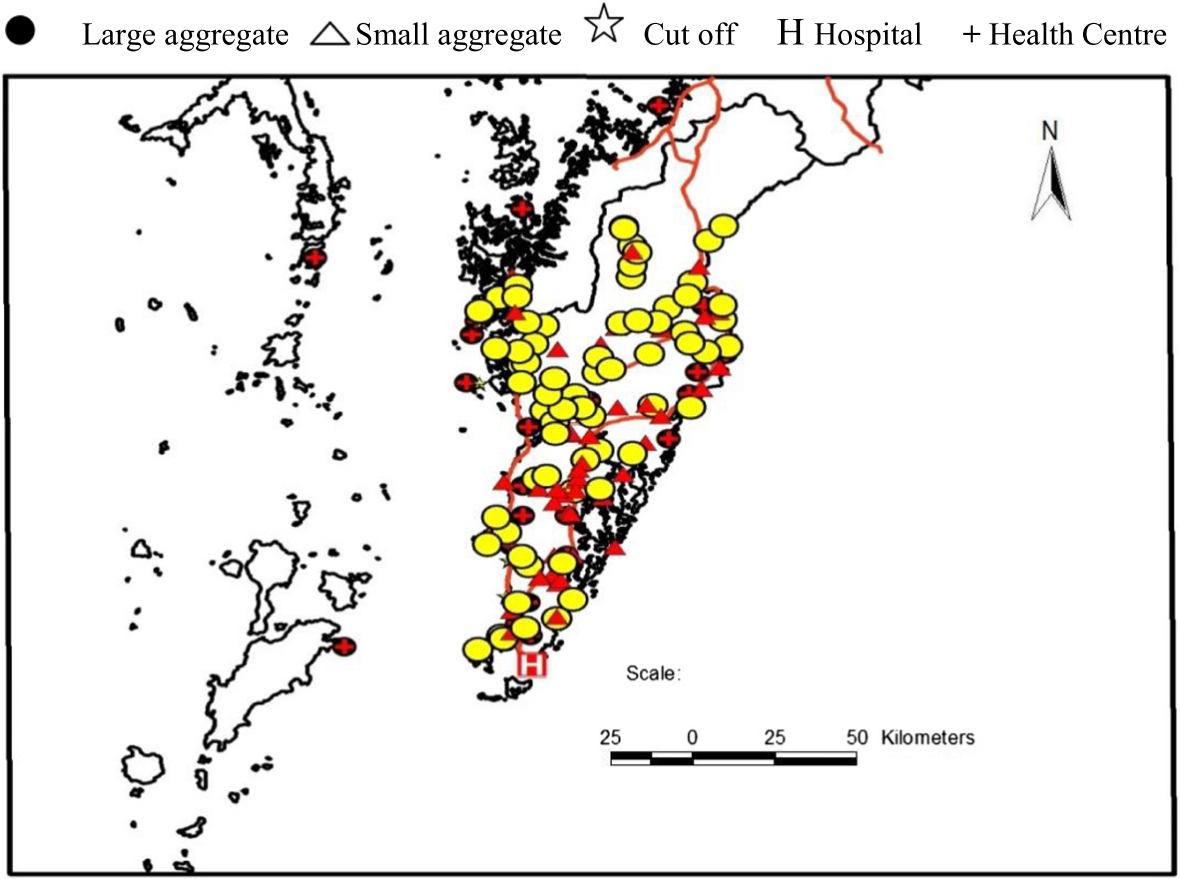
Spatial distribution of aggregates of temporary migrant workers and public health facilities (Kawthaung township).

The study was conducted from March to May in 2012, covering a total of 192 migrant aggregates, holding 28,174 temporary mobile/migrant workers and family members, and living in 8,018 households. Of these aggregates, 127 (66.1%) were located in Kawthaung township. The characteristics of study populations and aggregates are summarized in Table 1. More than 70% of the aggregates were identified as “large”. Around 87% of aggregates were in permanent places while 66.7% (8/12) of cut-off sites were temporary (see Figures 2 and 3). The ratio of migrant to permanent resident population in 192 aggregates was 0.66. Approximately half of the structure comprised male, aged ≥ 15 years and 57.7% of aggregates were occupied by palm oil plantation workers. Children under-five years contributed for 11.8% and night time forest dwellers were around 2,193, mostly in rubber plantations across all types of migrant aggregates (Table 1).

**Table 1:** Patterns of migration and structure, and access to health care for malaria by type of migrant aggregate

**Characteristic**	**Large aggregate**	**Small aggregate**	**Cut-off settlement**	**Total**
	**(n =102)**	**(n = 78)**	**(n = 12)**	**(n = 192)**
Pattern of migration				
Temporary place	12 (11.8)	6(7.7)	8 (66.7)	26 (13.5)
Permanent place	90 (88.2)	72 (92.3)	4 (33.3)	166 (86.5)
Estimated migrant households				
Sum of households	4,251	3,417	350	8,018
Median number of households	21.5	19.0	18.0	20.0
Range of households	4 - 500	4 - 710	9 - 100	4 -710
Estimated population of migrants	22,983	3,297	1,894	28,174
Ratio of migrants to permanent population	0.87	0.74	0.16	0.66
Population structure of migrants	(n = 22,983)	(n = 3,297)	(n = 1,894)	(n = 28,174)
<5 years old children	2,805 (12.2)	494 (15.0)	22 (1.2)	3,321 (11.8)
5-14 years old children	1975 (8.6)	391(11.9)	306 (16.2)	2672 (9.5)
≥ 15 years old male	11,868 (57.6)	1,523 (46.2)	1,275 (67.3)	14,666 (52.1)
≥ 15 years old female	6,335 (27.6)	889 (27.0)	291 (15.4)	7,515 (26.7)
Major economic activity	(n = 102)	(n = 78)	(n = 12)	(n = 192)
Palm oil plantations	62 (60.8)	45 (57.7)	0 (0.0)	107 (57.7)
Rubber plantations	18 (17.6)	19 (24.4)	0 (0.0)	37 (19.3)
Fishing	16 (15.7)	2 (2.6)	12 (100)	30 (15.6)
Cross-border, forest and mines	6 (5.9)	12 (15.4)	0 (0.0)	18 (9.4)
Night time forest dwellers	(n = 1,536)	(n = 657)	(n = 0)	(n = 2,193)
Palm oil plantations	535 (34.8)	217 (33.0)	0 (0.0)	752 (34.3)
Rubber plantations	801 (52.1)	310 (47.2)	0 (0.0)	1111 (50.7)
Cross-border, forest and mines	200 (13.1)	130 (19.8)	0 (0.0)	330 (15.0)

Percentages shown in parentheses.

Table 2 summarizes the nature of access to health care by study aggregates. All categories of migrant aggregates were located within an accessible distance to public health facilities, particularly sub-rural health center (RHC) (40.2% of large aggregates; 52% of small aggregates and 66.7% of cut-off settlements). The most common mode of transportation was motorcycle taxi, and the cost was about 1,500 kyat (approximately US$ 1.5) per person per one way travel within the mean duration of 30 minutes (Table 2). Approximately 14% (26/192) of study aggregates comprised of temporary mobile/migrant populations who traveled from Ayeyarwaddy and Yangon Regions, Mon (southern Myanmar), Rakhine (Western) or Shan (North-eastern) State. The majority of study respondents (72%) preferred to speak and understood Myanmar language in communication with health care providers in the study area. Common ethnic languages reported were Rakhine, Mon, Shan, and Kayin, and small minority reported to use dialects from other parts of Tanintharyi Region, and foreign languages (Malay and Thai). Malaria care was provided by basic health services of the public rural health centers.

**Table 2:** Access to health care for malaria by type of migrant aggregate

**Characteristic**	**Large aggregate**	**Small aggregate**	**Cut-off settlement**	**Total**
	**n =102**	**n = 78**	**n = 12**	**n = 192**
Nearest public health facility				
Township hospital	8 (7.8)	0 (0.0)	1 (8.3)	9 (4.8)
Station hospital	30 (29.4)	11 (14.7)	0 (0.0)	41 (21.7)
RHC	23 (22.5)	25 (33.3)	3 (25.0)	51 (27.0)
Sub RHC	41 (40.2)	39 (52.0)	8 (66.7)	88 (46.6)
Common mode of travel to the nearest public health facility				
On foot	70 (68.6)	47 (60.3)	8 (66.7)	125 (65.1)
Motorcycle	79 (77.5)	60 (76.9)	8 (66.7)	147 (76.6)
Bicycle	12 (11.8)	8 (10.3)	1 (8.3)	21 (10.9)
Boat	19 (18.6)	19 (24.4)	2 (16.7)	40 (20.8)
Car	42 (41.2)	24 (30.8)	0 (0.0)	66 (34.4)
Median cost (Myanmar kyat) of travel per person per one way				
Car	3000	500	0	1000
Motor cycle	2000	1000	500	1500
Boat	4000	4000	1150	4000
Mean duration (minute) of travel				
Car	21.9 ± 39.5	6.3 ± 18.3	0.0 ± 0.0	14.2 ± 32.1
On foot	118.5 ± 196.1	62.5 ± 95.8	29.6 ± 85.3	90.2 ± 159.4
Bicycle	5.3 ± 24.4	4.3 ± 16.7	1.3 ± 4.3	4.7 ± 20.7
Motorcycle	30.3 ± 32.7	19.3 ± 24.9	28.1 ± 85.7	25.7 ± 35.6
Boat	23.3 ± 96.9	13.5 ± 26.6	4.4 ± 11.4	18.1 ± 72.8
Malaria care providers				
Auxiliary midwife	14 (13.7)	1 (1.3)	1 (8.3)	16 (8.3)
Village practitioner	1 (1.0)	1 (1.3)	0 (0.0)	2 (1.0)
Malaria volunteer	5 (4.9)	9 (11.5)	2 (16.7)	16 (8.3)
Doctors from plantation sites	33 (32.4)	29 (37.2)	0 (0.0)	62 (32.3)
Basic health staff	49 (48.0)	38 (48.7)	9 (75.0)	96 (50.0)

=Percentages are shown in parentheses; Approximate conversion rate US$ 1 = 1000 kyat.

Table 3 reported household information and individual characteristics of temporary mobile/migrant workers. The majority (365/408; 89.5%) of migrants who were interviewed were identified as internal migrants, and only 10% interviewed were cross-border migrants, likely due to their clandestine nature of work. The frequency of reported border crossing was highest among palm oil plantation workers (58 times) and the lowest in fishing sites (5 times). Their mean duration of stay in the locality was 2.8 ± 1.1 years. Around 16% had intended to move out from this area within one year. Migrant households for any type of economic activity (type of work with earning) had mean number of 3.7 ± 1.9 members and 43.3% had earning capacity (engaged in livelihood with an income) with reported median family income per day as Kyat 5,000 (approximately 5 US Dollars). Male respondents prevailed (248/408, 60.8%) and 82.1% of them were married, while 78.2% stayed with their family members. Only 19.6% of migrant households reported night time work one week prior to the survey, mostly at fishing sites. Their average level of education was passed primary school (5.2 ± 2.5 years). Around 60% of households received pamphlets with malaria messages in Myanmar language. Bed net ownership was almost universal in which 55.6% was ITN/LLIN. Mostly, 26.8% of householders reported suspected malaria within past one year. Mobility patterns of temporary migrant workers linked to program operations for malaria control were cited during in-depth interviews as follows:

“Seasonal plantation workers here cross the border frequently and suffer from malaria most of the time. We need to prevent progress into severe stage because they reach health facilities sometimes after 4–5 days of fever.”

(Malaria volunteer, cross-border site)

“Some groups especially wood cutters from Rakhine State are highly mobile and lost within few weeks and reach Parchanriver on other side. They are difficult to cover by the malaria control program.”

(Doctor In-charge, Palm oil plantations)

**Table 3:** Social and demographic information of respondents by major economic activity

**Characteristic**	**Rubber**	**Fishing**	**Palm oil**	Others*	**Total**
	**n = 105**	**n = 54**	**n =183**	**n = 66**	**n = 408**
Nature of temporary migrant					
Internal migrant	91 (86.7)	48 (88.9)	181 (98.9)	45 (68.2)	365 (89.5)
Cross-border migrant	14 (13.3)	6 (11.1)	2 (1.1)	21 (31.8)	43 (10.5)
Intention to move outwithin one year	11 (10.5)	5 (9.3)	35 (19.1)	15 (22.7)	66 (16.2)
Household members					
Sum	371	221	665	261	1518
Mean ± SD	3.5 ± 1.8	4.1 ± 2.1	3.6 ± 1.8	4.0 ± 2.2	3.7 ± 1.9
Those with earning capacityǂ	169 (45.6)	82 (37.1)	288 (43.3)	118 (45.2)	657 (43.3)
Median daily family income (kyat)	6000	5000	4500	6000	5000
Nature of respondents					
Male respondent	56 (53.3)	40 (74.1)	106 (57.9)	46 (69.7)	248 (60.8)
Reported night time work	24 (22.9)	30 (55.6)	12 (6.6)	14 (21.2)	80 (19.6)
Average level of education (years)	5.1 ± 2.7	5.4 ± 2.3	5.1 ± 2.5	5.8 ± 2.4	5.2 ± 2.5
Owned bed nets in households	99 (94.3)	48 (88.9)	183 (100)	63 (95.5)	393 (96.3)
Number of bed nets owned	230	104	488	141	963
Proportion of ITNs/LLINs	135 (58.7)	47 (45.2)	280 (57.4)	73 (51.8)	535 (55.6)
Ratio of ITN/LLIN to householders	0.4	0.2	0.4	0.5	0.4
Suspected malaria (reported) in one year	87 (23.5)	38 (17.2)	212 (31.9)	70 (26.8)	407 (26.8)

Percentages shown in parentheses; *Cross-border, forest-goers, or odd jobs; Approximate conversion rate US$ 1 = 1,000 kyat; ǂThose who can make earning from their livelihood.

While the majority of respondents (sample of temporary migrant workers) were aware of fever, chills and rigor as malaria symptoms, headache was recognized only in a one-half of respondents. Approximately 60% knew to confirm malaria by microscopy as well as RDT and malaria medication differed by type of parasite found. A negligible proportion of the respondents could name antimalarial drugs. Knowledge of ITN as preventative of malaria in general, and the nets were given as priority for pregnant women was high, but it was not as high in recognizing the importance of protecting children and migrant workers using ITNs. A very few had knowledge of a link between malaria drug resistance and non-compliance to full course of antimalarials. Despite the apparently high level of knowledge in, and affordability to buy, ITNs (>80%), the proportion of respondents who reported the use of ITNs/LLINs the night before the survey and who were willing to buy them was disproportionately low (50-60%) (Table 4). Qualitative expressions of bednet use were provided below:

“It’s difficult for temporary migrant workers here to purchase LLIN. The places selling LLIN are far away from here and no NGOs for free distribution. It will be easy for them if they can receive sufficient numbers.

*(In-charge, rubber plantations, Pawei Island*)

**Table 4:** Knowledge, perceptions and practices related to EDPT and bed nets

**Characteristic**	**Rubber**	**Fishing**	**Palm oil**	**Others**	**Total**
	**n = 105**	**n = 54**	**n =183**	**n = 66**	**n = 408**
Knowledge of symptomsǂ					
Fever	71 (67.6)	42 (77.8)	131 (71.6)	45 (68.2)	289 (70.8)
Chills & rigor	89 (84.8)	46 (85.2)	146 (79.8)	51 (77.3)	332 (81.4)
Headache	63 (60.0)	17 (31.5)	85 (46.4)	36 (54.5)	201 (49.3)
Knowledge for malaria confirmationǂ					
Confirmed by microscopy	71 (67.6)	25 (46.3)	99 (54.1)	42 (63.6)	237 (58.1)
Confirmed by RDT	65 (61.9)	27 (50.0)	117 (63.9)	41 (62.1)	250 (61.3)
Awareness of antimalarialdrugsǂ					
Chloroquine	6 (5.7)	4 (7.4)	4 (2.2)	3 (4.5)	17 (4.2)
Artesunate	20 (19.0)	6 (11.1)	29 (15.8)	6 (9.1)	61 (15.0)
Quinine	4 (3.8)	3 (5.6)	5 (2.7)	2 (3.0)	14 (3.4)
Priority for ITNs/LLINsǂ					
Pregnant women	69 (65.7)	32 (59.3)	116 (63.4)	39 (59.1)	256 (62.7)
Under five children	97 (92.4)	40 (74.1)	167 (91.3)	61 (92.4)	365 (89.5)
Temporary migrant workers	37 (35.2)	11 (20.4)	78 (42.6)	25 (37.9)	151 (37.0)
Perceived risk of malaria	80 (76.2)	40 (74.1)	135 (73.8)	43 (65.2)	298 (73.0)
Perceived risk of non-compliance to full course of anti-malarialsǂ					
Parasites remained	51 (48.6)	20 (37.0)	78 (42.6)	24 (36.4)	173 (42.4)
Febrile again	76 (72.4)	30 (55.6)	119 (65.0)	49 (74.2)	274 (67.2)
Drug resistance	13 (12.4)	6 (11.1)	11 (6.0)	10 (15.2)	40 (9.8)
Sought help from public facilities	63 (60.0)	21 (38.9)	111(60.7)	35 (53.0)	230 (56.4)
Bed net use last night	n = 371	n = 221	n = 665	n = 261	n = 1518
Slept under ordinary net	118 (31.8)	86 (38.9)	189 (28.4)	107(41.0)	500 (32.9)
Slept under ITN/LLIN	208 (56.1)	95 (43.0)	419 (63.0)	122 (46.7)	844 (55.6)
To buy ITNs/LLINsǂ	n = 105	n = 54	n =183	n = 66	n = 408
Willingness	95 (90.5)	46 (85.2)	164 (89.6)	56 (84.8)	361 (88.5)
	n = 70	n = 30	n = 154	n = 40	n = 294
Affordability	46 (65.7)	19 (63.3)	80 (51.9)	32 (80.0)	177(60.2)

Percentages shown in parentheses; ǂSingle item response.

Nearly 77% preferred trained volunteers but just over half of respondents preferred channeling information, education and communication (IEC) messages by collaboration and partnership of authorities concerned. Only 32.4% of respondents across all clusters (lowest in palm oil plantations) realized the importance of community participation in malaria prevention and control (Table 5). In-depth interviewees from rubber plantations and fishing sites expressed their preferences:

“We have so many constraints to confirm malaria. We prefer information on RDT, skills to use RDT and to give appropriate antimalarials within 24 hours.”

(In-charge, rubber plantations, Kawthaung)

“We need collaborative work between Health Department and Administrators to inform and motivate the regular use of LLIN.”

(Employer, fishing site, Bokepyin)

**Table 5:** Preferences of temporary migrant workers for strengthening collaboration in malaria prevention and control

**Characteristicǂ**	**Major economic activity**				**Total**
	**Rubber**	**Fishing**	**Palm oil**	**Others**	
	**(n = 105)**	**(n = 54)**	**(n =183)**	**(n = 66)**	**(n = 408)**
Trained volunteers	84 (80.0)	36 (66.7)	141 (77.0)	52 (78.8)	313 (76.7)
Channeling IEC by collaboration	58 (55.2)	29 (53.7)	106 (57.9)	25 (37.9)	218 (53.4)
Partnership of authorities concerned	60 (57.1)	24 (44.4)	85 (46.4)	39 (59.1)	208 (51.0)
Arrangement of local funds	43 (41.0)	23 (42.6)	92 (50.3)	32 (48.5)	190 (46.6)
Organizing community participation	40 (38.1)	16 (29.6)	46 (25.1)	30 (45.5)	132 (32.4)

Percentages are shown in parentheses; ǂSingle item response.

## Discussion

Within the diverse aggregates of temporary mobile/migrant workers in Kawthaung and Bokepyin townships (Figures 2 and 3), the working hours of adult migrant workers including women varied with the type of economic activities. Transportation constraints, as well as severe weather and security concerns particularly at night, hampered the workers’ access to formal health services for EDPT. Even though some study aggregates were within the reach of malaria care providers and the public health facilities (Figures 2 and 3), their frequency of visits to the assigned migrant aggregates was unknown, and a high mobility of some temporary aggregates (e.g. fishing communities who moved every 2–3 months) may make the provider visit difficult. The distribution mechanisms of commodities such as ITNs/LLINs, rapid diagnostic test (RDT), and artemisinin combination therapy (ACT) to these aggregates through public health facilities required special attention. Replacement of oral artemisinin-based monotherapy in market with subsidized ACT and RDT through social marketing was designated as one of the key activities of NMCP [9]. We found that there were many multilingual ethnic groups in the region, and the distribution of health education messages only in Myanmar language may lead to limited understanding and receptiveness. Although the majority of the migrant populations understood Myanmar language, limited education level may impede their understanding of the message [11]. As shown in other studies conducted in similar circumstances [8,14-16], these physical, social and language barriers in the study population likely increase the potential risk in malaria transmission and spread of artemisinin resistance. Effective communication in this diverse high-risk group is critical and may be better reached by the use of simple language, inclusion of common ethnic languages in addition to Myanmar, and/or well-trained motivated interpreters.

About a one-half of the study population did not know that malaria should be diagnosed using microscopy or RDT, and only negligible number could name the recommended malaria drugs. This observation is consistent with the previous finding during the MARC survey [1]. As observed in other studies [16], self-medication practices were common, andthe private sector was typically preferred over the public sector. The majority of the migrant workers reported to rely on cocktail mixtures of drugs or artemisinin monotherapy, rather than prescribed ACT, freely available in the unregulated private sectors. The limited availability of ACT has been seen in the past due to inconsistent supplies and stock-outs, as reported in MARC Health Facility Survey in 2012 [1]. However with a highly successful marketing of subsidized ACT, these events are likely to be rare.

Interestingly there is a significant discrepancy in knowledge, attitude and practice of ITNs/LLINs. Apparently two nets per 5 persons was reported to be inadequate. Despite good knowledge of ITNs as protective measure of malaria, the reported rate of ITN use was low. Many of the migrant workers were outdoor night-time workers (e.g. plantation workers, wood cutters, rubber tappers, rat-catchers, etc.), and they did not use ITNs/LLINs, consistent with previous findings in Thailand [17]. Innovative methods of personal protection and behavioral change models are needed to optimize the use of ITNs/LLINs in the high-risk populations.

Currently, the non-governmental organizations (NGOs) working for malaria in villages/migrant aggregates in study townships included Myanmar Medical Association, Population Services International, and World Vision, Myanmar. Myanmar is the third country to initiate multilateral actions against artemisinin resistance in the Greater Mekong Sub-region, with support from Department for International Development (DFID) [18]. Our finding indicated that the majority of the respondents preferred volunteers (>75%) to strengthen prevention and control measures against malaria and over 50% preferred to channel information, education and communication (IEC) messages through collaboration in the locality. Findings from our study and others highlighted the need to improve the mechanisms of communication and coordination among multiple partners engaged in artemisinin resistance [8,9]. Potential strategies to maximize accessibility to malaria interventions may include the following: 1) tracking of foci of infections in mobile populations and mapping of the extent and distribution of malaria infections [19]; 2) real-time frequent sharing of information on drug resistant malaria; 3) frequent update of counterfeit and sub-standard antimalarial drug use; 4) setting up contact screening points for improved availability and use of RDTs and optimal use of quality antimalarial drugs.

## Conclusion

Migrant aggregates are geographically widely scattered and have a limited access to appropriate malaria knowledge and quality malaria care. The limitation may be caused by physical or logistical constraints, or social, linguistic or cultural barriers. Discrepancies between the knowledge and practices for malaria prevention and treatment indicated a serious need for further intensification in behavioral change, as well as innovative methods of protection that are attractive and convenient for the population. Unregulated and inappropriate use of artemisinin may lead to an increase in drug pressure and further fuel the development of artemisinin resistance. Artemisinin resistant malaria in the population with a high mobility carries a high risk of spread. A well-coordinated strengthened partnership of multiple stakeholders including employers of concern, public health workers, private medical practitioners, and implementing NGOs is urgently need to enhance the feasibility of appropriate interventions including transparent dissemination of information. Community-based innovative approaches are desirable for relaying information related to ITNs/LLINs, RDT and ACT, and drug resistance within the social and economic diversity of migrant aggregates.

## Competing interests

The authors declare that they have no competing interest.

## Authors’ contributions

KTW, MPK, TO, and TTK prepared the study design, field work and analysis plan for this paper. MPK, PTZ and MHN were involved in field data collection and project management. KTW, MPK and TO wrote the paper and performed the data management and statistical analysis together with MTD, PTZ, and MHN. All authors have reviewed and approved the final manuscript.

## Pre-publication history

The pre-publication history for this paper can be accessed here:

http://www.biomedcentral.com/1471-2458/14/463/prepub

## Supplementary Material

Additional file 1 Questionnaire. Click here for file

Additional file 2 Migrant mapping. Click here for file
