# Supplementary material for: Spatial distribution, work patterns, and perception towards malaria interventions among temporary mobile/migrant workers in artemisinin resistance containment zone
Source: BMC Public Health. 2014 May 17;14:463. doi: 10.1186/1471-2458-14-463 (PMC4032392; doi:10.1186/1471-2458-14-463)
Supplement: Additional file 1 — Questionnaire. [file 1471-2458-14-463-S1.docx]

**Spatial distribution, work patterns, and perception towards malaria interventions among temporary mobile/migrant workers in artemisinin resistance containment zone**

Date of interview /__/__/ /__ /__ / /__/ /__/ Start time: ____ : ____

**SECTION A: Social and demographic characteristics and malaria experience**

| **S.N** | **Questions** | **Code** |  | | **Skip to** |
| --- | --- | --- | --- | --- | --- |
| A1. | Township | Kawtthaung  Bokepyin | 1  2 | |  |
| A2. | Village |  |  | |  |
| A3. | What is your permanent address? (State/Region) |  |  | |  |
| A4. | How long is your current stay in this place? (months)  **STOP INTERVIEW IF LESS THAN ONE MONTH STAY** |  | \|__\|__\| | |  |
| A5. | Type of temporary migrant work mostly engaged | Temp internal  Temp cross-border | 1  2 | |  |
| A6. | If you are a cross-border migrant, how frequently have you crossed within past 6 months? |  | \|__\|__\| | |  |
| A7. | Type of work currently engaged by the temporary migrant worker | Rubber  Fishing  Palm oil  Factory  Restaurant  Shop  Others---------------- | 1234567 | |  |
| A8. | Sex of the respondent | Male  Female | 1  2 | |  |
| A9. | Age of the respondent­­ (Age in completed years) |  | \|__\|__\| | |  |
| A10. | Have you had formal schooling? | Yes  No  Read and Write  Monastic education  Others ______________ | 1  2  3  4  5 | | A20 |
| A11. | If the respondent has formal schooling, what is the highest grade he/she passed? | \|__\|__\|  Passed KG  Not Passed KG | 98  99 | |  |
| A12. | Marital status of the respondent | Currently married  Single  Separated/Divorced  Widower | 1234 | |  |
| A13. | Are you accompanied by other family members during the current stay? | Yes  No | 1  2 | |  |
| **S.N** | **Questions** | **Code** | |  | **Skip to** |
| A14. | Number of family members of temporary migrant | If no other members, **fill 99** | | \|__\|__\| |  |
| A15. | Number of family members (apart from the respondent) who can make earning | If no other members, **fill 99** | | \|__\|__\| |  |
| A16. | Average daily family income (in kyat) | \|__\|__\|\|__\|__\|\|__\|__\| | |  |  |
| A17. | Have you or anyone in your family members been working at night? | Yes  No | | 1  2 | 🡪**A19** |
| A18. | If you or anyone in the family has been working at night, what are your/his/her average working hours across a week? |  | | \|__\|__\| |  |
| A19. | When is the common starting time of night work? |  | | \|__\|__\| |  |
| A 20. | When is the common finishing time of night work? |  | | \|__\|__\| |  |
| A21. | Number of adults who had fever suspected of malaria during past 6 months of current stay |  | | \|__\| |  |
| A22. | Number of children (<5 yrs) who had fever suspected of malaria during past 6 months of current stay | If no under five-fill in 8  If no fever- fill in 9 | | \|__\| |  |
| A23. | Have health personnel or volunteers visited your family to give health education during past ONE month of current stay? | Yes  No  Don’t know | | 1  2  3 |  |
| A24. | Have you ever received LLIN/ITN and pamphlets concerning malaria and its prevention & treatment during current stay? | Yes  No  Don’t know | | 1  2  3 |  |

**Section B: Facts about bed nets**

| **S.N** | **Questions** | **Code** |  | **Skip to** |
| --- | --- | --- | --- | --- |
| B1. | Do you have any mosquito net in your family? | Yes  No | 1  2 |  |
| B2. | If yes, number of mosquito net |  | \|__\|__\| |  |
| B3. | Is LLIN/ITN included among those nets? | Yes  No | 1  2 | Skip B10 |
| B4. | IF yes, number of LLIN/ITN |  | \|__\|__\| |  |
| B5. | How many times do you usually wash LLIN/ITN in a year? ( If not washed, fill in 99) |  | \|__\|__\| |  |
| B6. | Have you received LLIN/ITN free of charge? | Yes  No | 1  2 |  |
| B7. | If you received FOC, what was the source? | RHC  NGOs  Others(specify) ------ | 1  2  3 |  |
| B8. | If you did not receive FOC, can you afford to buy one? | Yes  No | 1  2 |  |
| B9. | Have you ever received tablets or being briefed for retreatment of LLIN/ITN? | Yes  No | 1  2 |  |
| B10. | It is necessary to comply guidelines provided by health staff/volunteer on how to use LLIN/ITN | Agree  Uncertain  Disagree  DK | 1  2  3  4 |  |
| B11. | Why don’t you have LLIN/ITN? | Not afford to buy one  Dislike  Not necessary  No specific health improvement  Others (specify) _______ | 1  2  3  4  5  6 |  |
| B12. | If you have no LLIN/ITN, are you willing to buy? | Yes  No | 1  2 |  |
| B13. | Do you know any place where you can get LLIN/ITN either FOC or buy? | Yes  No | 1  2 |  |
| B14. | If YES, please tell me those places. | Market place/Shop  Rural Health Center/ Sub-center  NGO  Other ( specify) _______ | 1  2  3  4 |  |
| B15. | At what season you usually use mosquito net? | Wet season only  Wet and cool seasons  Cool season only  All seasons  Whenever feeling cold  Whenever there is mosquito bite  Never  Other ( specify) _______ | 1  2  3  4  5  6  7  8 |  |
| B16. | What is your bedtime during past one week? | Before 9 pm  9-11 pm  After 11 pm  Not regular | 1  2  3  4 |  |
| B17. | No. of family members slept under untreated bed net  B17.1 Past one week  B 17.2 Last night |  | \|__\|__\|  \|__\|__\| |  |
| B18. | Number of family members slept under LLIN/ITN  B 18.1 Past one week  B 18.2 Last night |  | \|__\|__\|  \|__\|__\| |  |
| B19. | If everyone did not sleep with untreated or LLIN/ITN what were the reasons?  CIRCLE MORE THAN ONE | Insufficient bed nets  Not necessary  Night time work  Others (specify) _______ | 1  2  3  4 |  |
| B20. | Sleeping under LLIN/ITN can prevent malaria. | Yes  No  Don’t know | 1  2  3 |  |
| B 21. | Person (s) who should be given priority for sleeping under LLIN/ITN  CIRCLE MORE THAN ONE | Pregnant women  Under 5 children  Temporary migrant families  Others (specify) _______ | 1  2  3  4 |  |
| B22. | Sleeping under ITN can prevent 100% from the bite of mosquitoes carrying malaria parasite. | Agree  Disagree  Undecided  Don’t know | 1  2  3  4 |  |
| B23. | If you are bitten by mosquitoes between dusk to dawn, you might have a chance to contract malaria. | Yes  No  Don’t know | 1  2  3 |  |
| B24. | Do you think that you have a chance of being infected by malaria? | Yes  No  Uncertain  Don’t know | 1  2  3  4 |  |
| B25. | Can you tell me how to treat the bed net? | Yes  No | 1  2 |  |
| B26. | From whom/where you got information regarding the use of LLIN/ITN for prevention of malaria?  CIRCLE MORE THAN ONE | One of the family members  Friends  Neighbors  Relatives  Health personnel  IEC materials  Village authorities  Border check points  Employers  Others (specify) | 1  2  3  4  5  6  7  8  9  10 |  |

**SECTION C: Knowledge and experience of malaria, early diagnosis and treatment**

| **S.N** | **Questions** | | **Code** | | |  | **Skip to** |
| --- | --- | --- | --- | --- | --- | --- | --- |
| C1. | What are the symptoms of malaria  CIRCLE MORE THAN ONE | | Fever  Chills  Headache  Sweating  Ache and pain  Coughing Others (specify) _______  Don’t know | | | 1  2  3  4  5  6  7  8 |  |
| C2. | If you or other temporary migrants have fever suspected of malaria what kind of medication do you/they usually try? | | Analgesics  Burmeton  Chloroquine  Quinine  Fansidar  Artesunate  Others (specify)-------------- | | | 1  2  3  4  5  6  7 |  |
| C3. | Do you know where to go if you don’t get better by self-medication for fever suspected of malaria? | | Yes  No | | | 1  2 |  |
| C4. | If YES, tell me about those places.  CIRCLE MORE THAN ONE | | Sub-center  RHC  Station Hospital  Township Hospital  GP clinic  Private Hospital  Other (specify) _______ | | | 1  2  3  4  5  6  7 |  |
| C5. | Do you know what is normally done in those places?  CIRCLE MORE THAN ONE | | Check and give anti-malarials  Give anti-malarials after checking for blood MP  Give anti-malarial after RDT  Others (specify) _______  Don’t know | | | 1  2  3  4  5 |  |
| C6. | | From where and whom you get information relating to early diagnosis and prompt treatment of malaria?  CIRCLE MORE THAN ONE | One of the family members  Friends  Neighbors  Relatives  Health personnel  IEC materials  Village authorities  Border check points  Employers  Other (specify) _______ | | | 1  2  3  4  5  6  7  8  9  10 |  |
| C7.  C 7.1  C 7.2 | | Do you know how to confirm malaria?  Blood film  Rapid diagnostic test | **Yes** | **No** | **DK** |  |  |
|  |  |  | 1 | 2 | 9 |  |  |
|  |  |  | 1 | 2 | 9 |  |  |
| C8. | | Type of malaria medication differs with type of malaria parasite | Yes  No  Don’t know | | | 1  2  3 |  |
| C9. | | When did you last go to the places stated in C4 with fever suspected of malaria? | ________ (month) \|__\|__\|__\|  **Never visited fill 999** | | |  |  |
| C10. | | What were the services provided during your last visit? | Check and give anti-malarials  Give anti-malarials after checking for blood MP  Give anti-malaria after RDT  Others (specify) _______  Don’t know | | | 1  2  3  4  5 |  |
| C11. | | Have you been informed about the results of checking blood? | Positive for malaria parasite  Negative for malaria parasite  Not informed | | | 1  2  3 |  |
| C12. | | Who referred you to that place?  CIRCLE MORE THAN ONE | One of the family members  Friends  Neighbors  Relatives  Health personnel  Village authorities  Border check points  Employers  Others (specify) _______ | | | 1  2  3  4  5  6  7  8  9 |  |
| C13. | | Have you received any medication for malaria prescribed by doctor/nurse/BHS? | Yes  No | | | 1  2 |  |
| C14. | | If YES, please cite the specific medication. |  | | |  |  |
| C15. | | People prone to more severe symptoms when contracting malaria infection  CIRCLE MORE THAN ONE | Pregnant women  Under 5 children  Non-resident/visitors  Temporary migrant workers  People with low resistance  Those with anti-malarial resistance  Others (specify) _______ | | | 1  2  3  4  5  6  7 |  |
| C16. | | Do you think it is necessary to follow instruction of health personnel to complete malaria treatment? | Yes  No  Uncertain  Don’t know | | | 1  2  3  4 |  |
| C17. | | If you do not comply to instructions of health personnel in treatment of malaria, what are the likely things that you might have encountered?  CIRCLE MORE THAN ONE | Nothing  Parasite remains in the body  Patient will continue to transmit malaria  Parasite will become resistant  Patient gets sick again  Patient does not recover  Others (Specify):___  Don't know | | | 1  2  3  4  5  6  7  8 |  |
| C18. | | Have you ever heard about drug resistant malaria in this region? | Yes  No  Don’t know | | | 1  2  3 |  |
| C19. | Which 3 activities do you think are the most important for the migrant workers to use LLIN/ITN and to know more about the early diagnosis and treatment of malaria?  CIRCLE 3 ACTIVITIES | | Partnership of authorities, BHS, NGOs, employers  Volunteers  Arrangement for local funds  Donation for required materials  Organizing for community participation  Channeling IEC by collaboration  Others (Specify)_____________ | | | 1  2  3  4  5  6  7 |  |

Interviewer ______________ End time ____ : _____
