# Supplementary material for: Spatial distribution, work patterns, and perception towards malaria interventions among temporary mobile/migrant workers in artemisinin resistance containment zone
Source: BMC Public Health. 2014 May 17;14:463. doi: 10.1186/1471-2458-14-463 (PMC4032392; doi:10.1186/1471-2458-14-463)
Supplement: Additional file 2 — Migrant mapping. [file 1471-2458-14-463-S2.pdf]

Migrant Mapping – Malaria
MARC Project - Mon State

MARC Tool no.1

Township – ....
Village Tract -......................................

Name of RHC........................
Name of sub-RHC...................................

Place of mapping (Tick only one)- A Village / Ward - ☐ A Cluster outside the village /ward- ☐

Place Name	GPS coding of the place		
....	Place........................	EO	N ....

1. Select the type of cluster / settlement as per defination provided. (Applicable only to a cluster)

1.1. Large cluster? ☐	1.2. Small cluster? ☐	1.3. Cut-off village settlement? ☐
• = & >60 people<br>• Far away from villages<br>• Homogenous in occupation<br>• Rubber plantation, Mine, Palm oil plantation	• < 60 people (25)<br>• Need not to be close to village<br>• Close to place of economic action<br>• Construction sites (Buildings, Dams, Roads)	• Close to village<br>• Share same resources with village<br>• Economically dependent on village<br>• Pond construction, Farming

2. Migration in details (Applicable only to cluster / settlement)

2.1. Pattern of migration (select one) Is it a temporary place? ☐ Is it a permanent place? ☐

2.2. Source - the place from where most of people of this cluster moved in-

a) ....State/Division..........................Township..........................village/ward

b) ....State/Division..........................Township..........................village/ward

**2.3. How long has this cluster been here?**	.........months	...............years
**2.4. How long will this cluster be here? (For a temporary cluster)**	.........months	...............years

2.5. Where does this cluster intent to move to a newer place? (only For a temporary cluster)

..........................State/Division..........................Township..........................village/ward

3. Occupation details

Common occupation		Common working hours	
		From	To
3.1			
3.2			

4. Language for communication

	Language	Ability	
		Speaking	Reading
4.1	Myanmar		
4.2	Other (....................)		

5. Total population ....

	Age Group	Permanent (Village)		Migrant/Mobile (Village / Cluster / Cut off Village settlement)	
		M	F	M	F
5.1	Under 5 years				
5.2	5 to 14 years				
5.3	15 years & above				
	Total				

6. No. of Forest dwellers

Estimated no. of people working at night time in forest (Forest dweller)
....

7. Households details (Village / Cluster / Settlement)

7.1. No. of houses	7.2. No. of households

8. Accessibility and availability of health facility and services close to this location

8.1. Which is the nearest public health facility (Tick only one) Sub-RHC ☐ RHC ☐ Station hospital ☐ Township hospital ☐

Name of that health facility..

8.2. Means of accessibility to the above mentioned public health facility (Answer all if applicable)

Type of transport	Duration		Estimated cost (One way)	Is there any seasonal barrier?	
	Hours	Minute		Yes	No
1. By car					
2. By motor bike					
3.By bicycle					
4. By foot					
5. By boat					

8.3. Place and category of any malaria service provider for this location.

a) Tick the most appropriate

AMW ☐ TTBA ☐ Village Practitioner ☐ Pharmacy ☐ Malaria Volunteer ☐ Dr. ☐ Other ☐

b) Provide more information about the service provider

Name... Job category.......................

Name... Job category.......................

9. Knowledge and practice of Malaria

9.1. How is Malaria transmitted?

a).. b)..

9.2. How do you prevent Malaria?

a).. b)..

10. Is there any other INGOs / NGOs organization currently working for Malaria diseases in this village or clusters? (Select one) Yes ☐ No ☐

If "Yes", please provide that INGOs / NGOs name- ....

11. Situational assessment (Tick the appropriate)

Ask the following questions		Yes	No
11.1.	Is this place stable?		
11.2.	Is it a restricted place?		

12. Operational coding (Tick the appropriate)

12.1. Normal operation	
12.2. Restricted operation	
12.3. Difficult operation	

Remark: Provide a line diagram for this location on the back of the sheet. (For cluster, please include information of a nearby village).

Name of surveyor - .... Title - .... Date - ....

Page- 2/2

***Version no.* 5 / 30th January 2012**
